# Preferences of orthopedic surgeons for treating midshaft clavicle fracture in adults

**DOI:** 10.1590/S1679-45082017AO4043

**Published:** 2017

**Authors:** Adilson Sanches de Oliveira, Bruno Braga Roberto, Mario Lenza, Guilherme Figueiredo Pintan, Benno Ejnisman, Breno Schor, Eduardo da Frota Carrera, Joel Murachovsky

**Affiliations:** 1 Hospital Israelita Albert Einstein, São Paulo, SP, Brazil.; 2 Escola Paulista de Medicina, Universidade Federal de São Paulo, São Paulo, SP, Brazil.; 3 Faculdade de Medicina do ABC, Santo André, SP, Brazil.

**Keywords:** Fractures bone/surgery, Fractures, bone/therapy, Clavicle/injuries, Clavicle/surgery, Surveys and questionnaires, Fraturas ósseas/cirurgia, Fraturas ósseas/terapia, Clavícula/lesões, Clavícula/cirurgia, Inquéritos e questionários

## Abstract

**Objective:**

To determine the current clinical practice in Latin America for treating midshaft clavicle fractures, including surgical and non-surgical approaches.

**Methods:**

A cross-sectional study using a descriptive questionnaire. Shoulder and elbow surgeons from the Brazilian Society of Shoulder and Elbow Surgery and from the Latin American Society of Shoulder and Elbow were contacted and asked to complete a short questionnaire (SurveyMonkey^®^) on the management of midshaft fractures of the clavicle. Incomplete or inconsistent answers were excluded.

**Results:**

The type of radiographic classification preferably used was related to description of fracture morphology, according to 41% of participants. Allman classification ranked second and was used by 24.1% of participants. As to indications for surgical treatment, only the indications with shortening and imminence of skin exposure were statistically significant. Conservative treatment was chosen in cortical contact. Regarding immobilization method, the simple sling was preferred, and treatment lasted from 4 to 6 weeks. Although the result was not statistically significant, the blocked plate was the preferred option in surgical cases.

**Conclusion:**

The treatment of midshaft clavicle fractures in Latin America is in accordance with the current literature.

## RESUMO

**Objetivo:**

Determinar a prática clínica atual na América Latina para o tratamento das fraturas do terço médio da clavícula, incluindo abordagens cirúrgicas e não cirúrgicas.

**Métodos:**

Estudo transversal com aplicação de questionário descritivo. Cirurgiões de ombro e cotovelo da Sociedade Brasileira de Cirurgia do Ombro e Cotovelo e da Sociedade Latino-Americana de Ombro e Cotovelo foram contatados e convidados a completar um breve questionário (SurveyMonkey^®^) sobre o manejo das fraturas do terço médio da clavícula. Foram excluídas as respostas incompletas ou inconsistentes.

**Resultados:**

O tipo de classificação radiográfica utilizada de preferência esteve de acordo com a descrição da morfologia da fratura, representando 41% do total dos participantes. Em segundo lugar, apareceu a classificação de Allman, que foi utilizada por 24,1% dos participantes. Nas indicações de tratamento cirúrgico, as indicações com encurtamento e iminência de exposição da pele foram estatisticamente significativas. Tratamento conservador foi prescrito em caso de contato entre as corticais. Como método de imobilização, a tipoia simples foi a preferência, e o tempo de tratamento foi de 4 a 6 semanas. Apesar do resultado sem significância estatística, a placa bloqueada foi a opção preferencial nos casos cirúrgicos.

**Conclusão:**

A metodologia de tratamento das fraturas do terço médio da clavícula nos países da América Latina é semelhante, assim como com a literatura atual.

## INTRODUCTION

Clavicle fractures are considered common and represent 2.6 to 4% of all fractures in adult population, and 35% of all shoulder girdle injuries.^(^ ^1^ ^)^ The incidence of this type of fracture among adolescents and adults is 29 and 64 per 100,000 per year, respectively.^(^ ^2^ ^)^

To better assess and treat this type of fracture, several classification systems were devised based on displacement and anatomical location.^(^ ^3^ ^-^ ^5^ ^)^ Most fractures occur on the midshaft of the clavicle (81%).^(^ ^6^ ^)^

Non-operative management (conservative treatment) is traditionally used to treat midshaft fractures without displacement, due to the low frequency of pseudoarthrosis.^(^ ^7^ ^,^ ^8^ ^)^ The most common treatment options are the use of a sling, a ‘figure-of-eight’ bandage/immobilization, or a combination of these two methods.^(^ ^9^ ^-^ ^11^ ^)^

Currently, some of the indications for operative treatment include exposed fractures, neurovascular involvement, skin involvement, displacement of bone fragments, initial shortening greater than 20mm, severe comminution, floating shoulder, and vicious consolidation/pseudarthrosis.^(^ ^12^ ^)^ The most commonly used operative approaches are open reduction and internal fixation with flexible plates or rods.^(^ ^13^ ^)^

At present, there are few randomized trials comparing surgical and conservative approaches in the treatment of clavicle fractures, and limited evidence from studies on the effectiveness of different surgical and non-operative methods for the treatment of clavicle fractures.^(^ ^14^ ^-^ ^16^ ^)^

As a first step in considering the development of clinical studies focused on effectiveness of different types of therapeutic approaches, we aimed to determine the current practice in the management of these fractures.

## OBJECTIVE

To determine the current clinical practice in Latin America for the treatment of midshaft clavicle fractures, including surgical and non-operative approaches.

## METHODS

The study was conducted at the *Hospital Israelita Albert Einstein* and according to the requirements of the National Health Council resolution number 466/2012. The study was initiated after approval by the Research Ethics Committee of the *Hospital Israelita Albert Einstein*, under the opinion number 1.047.385, CAAE: 44158715.0.0000.0071. This was a cross-sectional study using a descriptive questionnaire, in which shoulder and elbow surgeons from the Brazilian Society of Shoulder and Elbow Surgery and the Latin American Shoulder and Elbow Society were contacted and invited to complete a brief questionnaire on the management of midshaft clavicle fractures.

This contact was made by an e-mail containing a brief explanation of the research and an access link. Invitations were sent to 971 members of the Brazilian Society of Shoulder and Elbow Surgery, from July 1^st^, 2015 to August 5^th^, 2016, and 400 invitations were sent to members of the Latin American Shoulder and Elbow Society, in the period from April 4^th^, 2016 to May 14^th^, 2016.

The questionnaires were completed online, and the answers and the identity of the participants were kept confidential. An online survey tool was used (SurveyMonkey^®^). After receiving the survey responses, the questionnaire was finalized and data analyzed.

The questionnaire contained questions regarding the opinions of orthopedic surgeons, to identify the clinical practice of these specialists in the treatment of midshaft clavicle fractures. Initially, to identify the available instruments and evaluate beliefs and assumptions about the treatment of these fractures, a search was performed in MEDLINE (via PubMed) and EMBASE. The terms used for searching were: [‘(*Clavicle* [mh] OR clavic* [tw] *OR collarbone* [tw]) *AND* (*Fracture Healing* [mh] *OR Fracture Fixation* [mh] *OR Fractures, Bone* [mh] OR fracture* [tw] *OR pseudarthrosis* [mh] OR pseudoarthros* [tw] OR pseudarthros* [tw])’] in PubMed. The terms used in EMBASE were: [‘*clavicle*/, (clavic* *or collarbone*). tw, exp *Fracture Healing*/ *or* exp *Fracture Treatment*/ *or* exp *Fracture*/ or exp *Pseudarthrosis*/, fracture* or pseudoarthros* or pseudarthros*). [tw’].

We chose to standardize the answers using a clinical case model, to reduce doubts and biases that could arise during the completion of the research. We prepared a pilot questionnaire, according to the approaches and indications currently available, which was later evaluated and reviewed by the group of shoulder and elbow surgery at *Hospital Israelita Albert Einstein* (Appendixs 1 and 2 – Appendix 2 was translated into Spanish after approval by the Ethics Committee).

Shoulder and elbow specialists, members of the Brazilian Society of Shoulder and Elbow Surgery or of the Latin American Shoulder and Elbow Society, were included in the survey. Incomplete, inconsistent questionnaires, and those submitted after the survey was closed were excluded.

We assessed the aforementioned professionals’ opinions on interventions related to the treatment of midshaft clavicle fractures in adult patients, such as classification, treatment options, possible complications, among others specified in the annexes. The questionnaires were sent to all members of both Societies. Based on other invstigations conducted in this format, we expected a 30 to 70% return rate of completed questionnaires.^(^ ^17^ ^-^ ^20^ ^)^

After data collection, we investigated whether there were significant differences among the preferences found in different regions of Brazil and between Brazil and other Latin American countries.

All pieces of information gathered were described as absolute and relative frequencies, except for the years of work experience, which were described as median, interquartile range, and minimum and maximum values. The variables were described as absolute and relative frequencies. The associations among the variables were evaluated by the χ^2^ test or Fisher’s exact test. The analyses were performed using the Statistical Package for the Social Sciences (SPSS) at a significance level of 5%.^(^ ^21^ ^,^ ^22^ ^)^

## RESULTS

Out of a total of 971 invitations sent, 571 invitations were sent to members of the Brazilian Society of Shoulder and Elbow Surgery, with a return of 283 completed questionnaires, and 400 invitations were sent to members of the Latin American Shoulder and Elbow Society, with a return of 75 completed questionnaires. A total of 971 invitations were sent out, and the response rate was 36.8%.

We excluded 14 questionnaires from the survey, due to incomplete or inconsistent answers, and included a total of 344 questionnaires: 269 (78.2%) from the Brazilian Society, and 75 (21.8%) from the Latin American Shoulder and Elbow Society.

It was not possible to apply statistical tests to all variables, such as to evaluate the association between the group of professionals and the variables immobilization time for patients treated non-operatively, preferred synthesis for spiral fracture, preferred synthesis for complex fracture, most frequently used plate position, and recommended time of immobilization after surgery. These variables presented a large number of categories, and very small response frequencies were found in some categories, rendering inadequate the application of statistical tests.

The distribution of the countries where the 75 Latin American surgeons worked was as follows: 65.3% from Argentina, 4.0% from Bolivia, 9.3% from Chile, 9.3% from Uruguay, 4.0% from Paraguay, 2.7% from Venezuela, 1.3% from Colombia, 1.3% from Ecuador, 1.3% from Mexico, and 1.3% from Nicaragua.

As to 269 Brazilian surgeons, the regional distribution was 60.6% from the Southeast Region, followed by the South Region with 16.0%, Northeast Region with 13.0%, Central Western Region with 8.6%, and North Region with 1.9%.

**Figure 1:**
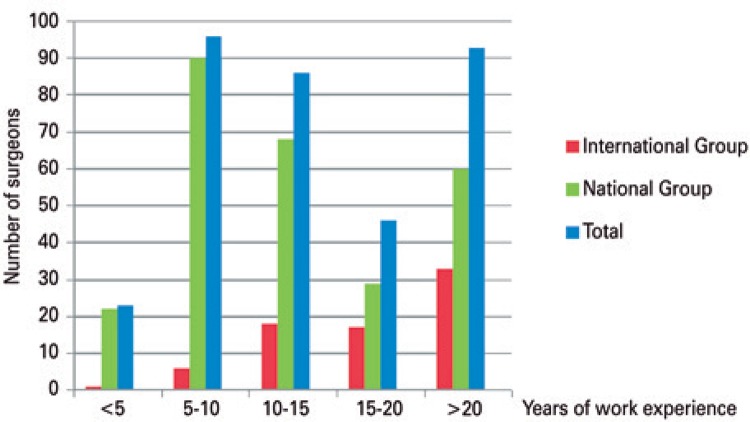
Years of work experience in orthopedics

In the evaluation of the participants’ work experience (Figure 1), we found that the majority of Brazilian orthopedic surgeons who answered the questionnaire had 5 to 10 years of work experience, accounting for 33.5% of the sample as compared to only 8% of foreign professionals. The most prevalent time of work experience among foreign physicians was over 20 years (44%) *versus* 22.3% of Brazilians, with p<0.001.

**Figure 2:**
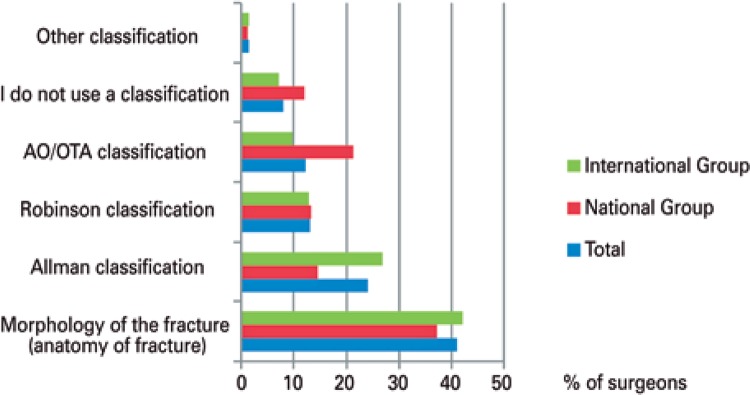
Preferred radiographic classification

Regarding the surgeon preferred type of radiographic classification, we obtained a result with statistical significance (p=0.03). Most Brazilian and foreign specialists use a classification system that describes the morphology of the fracture, representing 41% of total of participants. Allman system is used by 26.8% of Brazilian specialists and 14.7% of foreign specialists, accounting to 24.1% of total. The AO/OTA classification is used by 21.7% of foreign specialists and less used by Brazilian specialists (9.7%), totaling up 12.2% of the number of participants (Figure 2).

**Figure 3:**
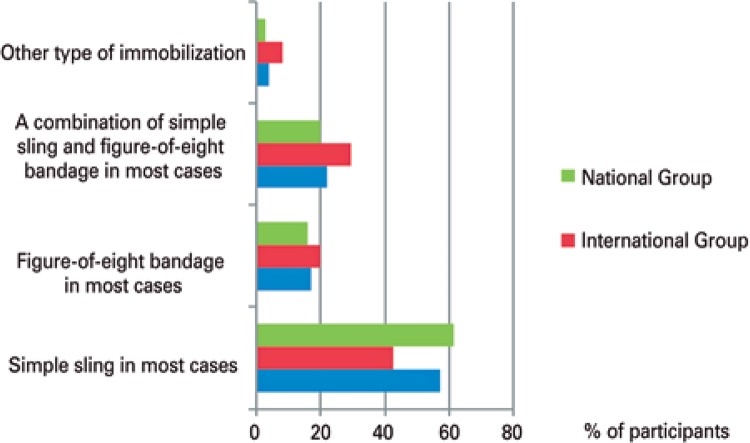
Type of immobilization for patients not submitted to surgery

**Figure 4:**
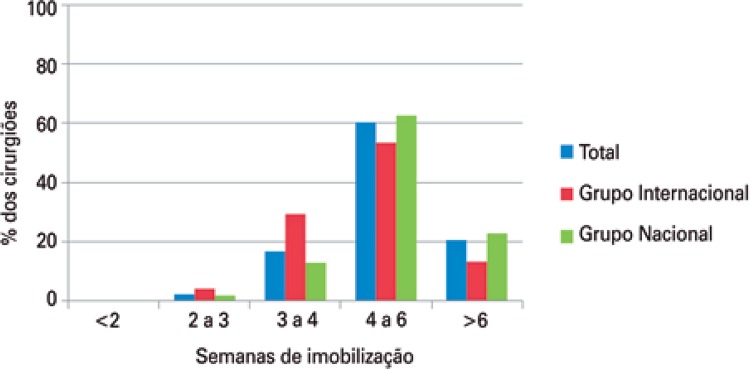
Treatment time with immobilizer for patients on conservative treatment

Conservative (non-operative) treatment of midshaft clavicle fractures for all patients, regardless of the type of fracture, was indicated by only 4.1% of participants, with statistically significant data (p=0.017), accounting for 9.3% of foreign surgeons, and 2.6% of Brazilian surgeons. Regarding immobilization in this type of treatment, the result was statistically significant (p=0.012). We found that most participants, both foreign and Brazilian, used only a simple sling as immobilization (57.2%), followed by a combination of simple sling and figure-of-eight bandage (22%), and by a figure-of-eight bandage alone (16.9%). As to time of immobilization, 60.4% of participating surgeons maintained it for 4 to 6 weeks (Figures 3 and 4).

The criteria for indication of surgical treatment are shown in table 1. In cases of displaced fracture, but with cortical contact, 88.2% of foreigners and 95.8% of Brazilians did not perform surgery and indicate only a conservative treatment. In fractures with shortening, 70.6% of foreigners and 84.7% of Brazilians indicated surgical treatment. When skin exposure is imminent, 91.6% of Brazilians and 60.3% of foreigners indicated a surgical approach, with no statistically significant differences in the other indications.

**Table 1:** Surgical indication according to parameter radiographic

	Total	Group		p value

		Latin American Society of Shoulder and Elbow Surgery	Brazilian Society of Shoulder and Elbow Surgery	
Displaced fracture with cortical contact				
No	310 (94.2)	60 (88.2)	250 (95.8)	p_2_=0.035
Yes	19 (5.8)	8 (11.8)	11 (4.2)	
Displaced fracture without cortical contact				
No	56 (17.0)	13 (19.1)	43 (16.5)	p_1_=0.606
Yes	273 (83.0)	55 (80.9)	218 (83.5)	
Fractures with shortening				
No	60 (18.2)	20 (29.4)	40 (15.3)	p_1_=0.007
Yes	269 (81.8)	48 (70.6)	221 (84.7)	
Comminuted fracture				
No	199 (60.5)	37 (54.4)	162 (62.1)	p_1_=0.250
Yes	130 (39.5)	31 (45.6)	99 (37.9)	
Segmental fracture				
No	184 (55.9)	37 (54.4)	147 (56.3)	p_1_=0.778
Yes	145 (44.1)	31 (45.6)	114 (43.7)	
Imminent skin exposure				
No	49 (14.9)	27 (39.7)	22 (8.4)	p_1_<0.001
Yes	280 (85.1)	41 (60.3)	239 (91.6)	
Evident clinical deformity (aesthetic aspect)				
No	232 (70.5)	47 (69.1)	185 (70.9)	p_1_=0.776
Yes	97 (29.5)	21 (30.9)	76 (29.1)	

**Figure 5:**
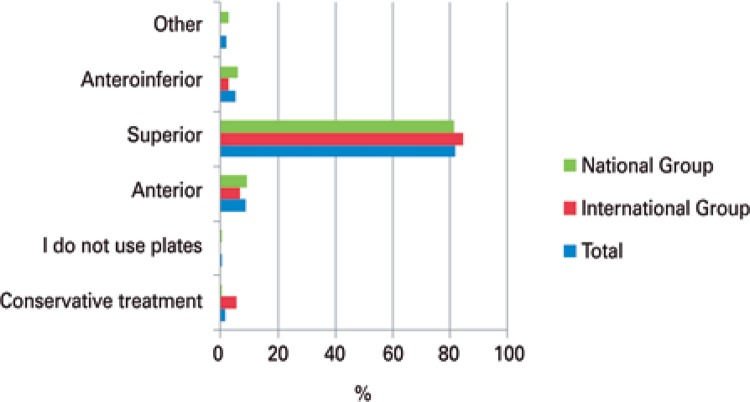
Position of the most often used plate

In the surgical treatment for transverse midshaft clavicle fractures, the preferred option was the use of a pre-contoured locking plate, with statistical significance. Although no statistical tests were conducted in some results, or no statistically significant results were obtained when they were applied, there was a higher preference for the use of pre-contoured locking plates in all types of fractures (Table 2), and in most cases, the preferred location was the superior aspect of the clavicle (Figure 5).

**Table 2:** Preferred synthesis

Types of fractures	Total	Group		p value

		Latin American Society of Shoulder and Elbow Surgery	Brazilian Society of Shoulder and Elbow Surgery	
Spiral fracture				
I only perform non-operative treatment	11 (3.3)	6 (8.2)	5 (1.9)	---
Kirschner wire	1 (0.3)	0 (0.0)	1 (0.4)	
Flexible intramedullary nail	2 (0.6)	0 (0.0)	2 (0.8)	
DCP plate	24 (7.1)	5 (6.8)	19 (7.2)	
LCP plate	9 (2.7)	2 (2.7)	7 (2.7)	
LC-DCP plate	15 (4.5)	4 (5.5)	11 (4.2)	
Pre-contoured locking plate	213 (63.2)	47 (64.4)	166 (62.9)	
Reconstruction plate	48 (14.2)	6 (8.2)	42 (15.9)	
One-third tubular plate	2 (0.6)	0 (0.0)	2 (0.8)	
Other type of surgical treatment	12 (3.6)	3 (4.1)	9 (3.4)	
Oblique fracture				
I only perform non-operative treatment	9 (2.7)	6 (8.2)	3 (1.1)	p_2_=0.059
Kirschner wire	7 (2.1)	1 (1.4)	6 (2.3)	
Flexible intramedullary nail	3 (0.9)	0 (0.0)	3 (1.1)	
DCP plate	26 (7.7)	4 (5.5)	22 (8.3)	
LCP plate	12 (3.6)	3 (4.1)	9 (3.4)	
LC-DCP plate	17 (5.0)	5 (6.8)	12 (4.5)	
Pre-contoured locking plate	215 (63.8)	44 (60.3)	171 (64.8)	
Reconstruction plate	35 (10.4)	5 (6.8)	30 (11.4)	
Other type of surgical treatment	13 (3.9)	5 (6.8)	8 (3.0)	
Transverse fracture				
I only perform non-operative treatment	12 (3.6)	8 (11.0)	4 (1.5)	p_2_=0.006
Kirschner wire	6 (1.8)	2 (2.7)	4 (1.5)	
Flexible intramedullary nail	10 (3.0)	3 (4.1)	7 (2.7)	
DCP plate	36 (10.7)	5 (6.8)	31 (11.8)	
LCP plate	11 (3.3)	4 (5.5)	7 (2.7)	
LC-DCP plate	33 (9.8)	9 (12.3)	24 (9.1)	
Pre-contoured locking plate	186 (55.4)	36 (49.3)	150 (57.0)	
Reconstruction plate	32 (9.5)	3 (4.1)	29 (11.0)	
One-third tubular plate	2 (0.6)	1 (1.4)	1 (0.4)	
Other type of surgical treatment	8 (2.4)	2 (2.7)	6 (2.3)	
Complex fracture				
I only perform non-operative treatment	7 (2.1)	4 (5.6)	3 (1.1)	---
Kirschner wire	1 (0.3)	0 (0.0)	1 (0.4)	
Flexible intramedullary nail	3 (0.9)	0 (0.0)	3 (1.1)	
DCP plate	8 (2.4)	2 (2.8)	6 (2.3)	
LCP plate	12 (3.6)	2 (2.8)	10 (3.8)	
LC-DCP plate	15 (4.5)	3 (4.2)	12 (4.6)	
Pre-contoured locking plate	237 (70.7)	48 (66.7)	189 (71.9)	
Reconstruction plate	38 (11.3)	8 (11.1)	30 (11.4)	
Other type of surgical treatment	14 (4.2)	5 (6.9)	9 (3.4)	

DCP: Dynamic Compression Plate; LCP: Locking Compression Plate; LC-DCP: Low-Contact Dynamic Compression Plate.

## DISCUSSION

Midshaft clavicle fracture is still a much discussed topic regarding its classification, type of treatment and relevant outcomes for evaluation. The classification system mostly used in Brazil is the same applied in other Latin American countries, *i.e.*, the descriptive classification of fracture morphology. Burnham et al.^(^ ^23^ ^)^ explained that this type of classification is the most relevant in the indication of surgical fixation. The authors also reported that the most accepted and most widely used classification in the world is Allman system, which ranked second in the preference of specialists participating in this research.^(^ ^23^ ^)^

In the past, treatment of midshaft clavicle fractures was traditionally conservative, with low rates of pseudarthrosis, but there were no studies comparing functional results with surgical treatment.^(^ ^7^ ^)^ Today, however, many researchers recommend surgical treatment for clavicle fractures, leading to better functional results, less pain after surgery, and early return to labor/sports activities.^(^ ^12^ ^,^ ^24^ ^,^ ^25^ ^)^ Nonetheless, the choice of treatment is not only limited by fracture characteristics, but also by expectations of treatment benefits, perceptions on risk factors for pseudarthrosis with conservative treatment, as well as by potential complications of surgical interventions. Furthermore, some variables, such as activity level and time to return to daily activities, should be considered when selecting treatment.

For the conservative treatment of the clavicle, the most used methods today are a simple arm sling and a figure-of-eight bandage. There are few studies comparing these types of immobilization. A study conducted by Andersen et al.^(^ ^9^ ^)^ showed that conservative treatment with a simple arm sling is more comfortable for the patient, but there was no significant difference in the functional results. This method of immobilization was the choice of US orthopedic surgeons in a study carried out by Heuer et al.^(^ ^26^ ^)^ In contrast, a study conducted by Pieske et al.,^(^ ^18^ ^)^ showed that a figure-of-eight bandage is the preferred method for treating clavicle fractures in that country. Stanley and Norris^(^ ^27^ ^)^ concluded that there was no significant difference between treatment with simple sling or figure-of-eight bandage. In the present study, the preference of surgeons was to use a simple sling (57.2%) as method of immobilization.

For the surgical treatment, several methods can be used, such as locking plate, flexible intramedullary nail, reconstruction plate, Kirschner wires, etc. Currently, there are many studies comparing different surgical methods, with similar results and no significant difference among methods, especially in the comparison of intramedullary pins with plates. Wang et al.,^(^ ^28^ ^)^ for instance, compared intramedullary nail *versus* plate and screw fixation. The authors showed that the two methods are equivalent, with no significant differences found in relation to complications, patient satisfaction and functional results, and they differ only in surgical time, which is shorter in the case of intramedullary nail. However, functional improvement is achieved more rapidly when treating with the plate. Zeng et al.,^(^ ^29^ ^)^ compared the use of flexible intramedullary nail with reconstruction plate and concluded that patients in the reconstruction plate fixation group showed earlier functional return. The reason was this fixation method establishes greater fracture stability despite of longer surgical time when compared to the flexible nail. In the present study, the preference of surgeons when indicating surgical treatment was the use of a pre-contoured locking plate, with a significant result only in transverse fractures.

There are different positions of plate placement in the synthesis of midshaft fracture clavicle. The most used positions are superior, anteroinferior, and anterior. Some studies showed that the anteroinferior position can reduce the risk of irritating symptoms caused by the synthetic material when compared to the placement of the plate in a superior position, due to the prominence of the implant. Besides reducing this risk, with the plate at an anteroinferior position, the screws are safely placed at a posterosuperior position, avoiding iatrogenic neurovascular lesions. In addition, with this position of the plate, the use of larger screws is feasible, allowing a better fixation.^(^ ^30^ ^)^ On the other hand, Celestre et al.,^(^ ^31^ ^)^ showed that the biomechanics of the plate positioned superiorly on the clavicle resulted in improved stability and less rigidity, as compared to the anteroinferior plate. However, there was no statistically significant result regarding plate position preference in our study.

## CONCLUSION

The clinical practice for managing clavicle fractures tends to surgery rather than conservative treatment. Both Brazilian and foreign surgeons in Latin America showed this different approach about this type of fracture.

## APPENDIX 1

**Table:** QUESTIONNAIRE FOR THE BRAZILIAN SOCIETY

Instructions:
Only one valid answer to each question, except question 5.
Consider the following conditions as a pattern for all questions.
**Treatment of CLOSED MIDSHAFT clavicle fractures in ADULT and CLINICALLY STABLE patients, with NO other fractures (excluding floating shoulder) or associated lesions (vascular and/or neurological)**
Years of work experience in orthopedics:___________
Region where you work in Brazil (State):
( ) Acre (AC)
( ) Alagoas (AL)
( ) Amapá (AP)
( ) Amazonas (AM)
( ) Bahia (BA)
( ) Ceará (CE)
( ) Distrito Federal (DF)
( ) Espírito Santo (ES)
( ) Goiás (GO)
( ) Maranhão (MA)
( ) Mato Grosso (MT)
( ) Mato Grosso do Sul (MS)
( ) Minas Gerais (MG)
( ) Paraíba (PB)
( ) Paraná (PR)
( ) Pernambuco (PE)
( ) Piauí (PI)
( ) Rio de Janeiro (RJ)
( ) Rio Grande do Norte (RN)
( ) Rio Grande do Sul (RS)
( ) Rondônia (RO)
( ) Roraima (RR)
( ) Santa Catarina (SC)
( ) São Paulo (SP)
( ) Sergipe (SE)
( ) Tocantins (TO)
3. Type of work:
( ) Associated with an education institution
( ) No association with education institution
4. What radiographic classification do you prefer to use?
( ) Fracture morphology (fracture anatomy)
( ) Allman classification
( ) Robinson classification
( ) AO/OTA classification
( ) I do not use any classification
( ) Other classification: _____________________________________
5. Which of the following criteria would lead you to indicate a surgical treatment? (THIS IS THE ONLY QUESTION THAT ALLOWS MORE THAN ONE ANSWER).
( ) All my patients are treated with NON-operative treatment (if you choose this alternative, go to question 6)
( ) All my patients are treated with surgical treatment (if you choose this alternative, go to question 6)
( ) Displaced fracture WITH cortical contact
( ) Displaced fracture WITHOUT cortical contact (>2cm)
( ) Fracture shortening (>2cm)
( ) Comminuted fracture (>3 fragments)
( ) Segmental fracture
( ) Imminence of skin exposure
( ) Significant clinical deformity (aesthetic deformity)
6. For patients who are NOT surgically treated, what type of immobilization do you use?
( ) I only perform surgical treatment
( ) I do not use any type of immobilizer
( ) Simple arm sling in most cases
( ) Figure-of-eight bandage in most cases
( ) A combination of sling and figure-of-eight bandage in most cases
( ) Other type of immobilization. Please specify below:
___________________________________________________________
7. For patients treated NON-operatively using immobilizer, for how long do you continue the treatment?
( ) I only perform surgical treatment
( ) Up to two weeks
( ) Two to three weeks
( ) Three to four weeks
( ) Four to six weeks
( ) More than six weeks
8. For surgical treatment, what is the preferred synthesis used in spiral fractures?
( ) I only perform NON-operative treatment
( ) Kirschner wire
( ) Flexible intramedullary nail
( ) DCP plate
( ) LCP plate
( ) LC DCP plate
( ) Pre-contoured locking plate
( ) Reconstruction plate
( ) One-third tubular plate
( ) Other type of surgical treatment. Please specify below:
___________________________________________________________
9. For surgical treatment, what is the preferred synthesis used in oblique fractures?
( ) I only perform NON-operative treatment
( ) Kirschner wire
( ) Flexible intramedullary nail
( ) DCP plate
( ) LCP plate
( ) LC DCP plate
( ) Pre-contoured locking plate
( ) Reconstruction plate
( ) One-third tubular plate
( ) Other type of surgical treatment. Please specify below:
___________________________________________________________
10. For surgical treatment, what is the preferred synthesis used in transverse fractures?
( ) I only perform NON-operative treatment
( ) Kirschner wire
( ) Flexible intramedullary nail
( ) DCP plate
( ) LCP plate
( ) LC DCP plate
( ) Pre-contoured locking plate
( ) Reconstruction plate
( ) One-third tubular plate
( ) Other type of surgical treatment. Please specify below:
___________________________________________________________
11. For surgical treatment, what is the preferred synthesis used in complex fractures?
( ) I only perform NON-operative treatment
( ) Kirschner wire
( ) Flexible intramedullary nail
( ) DCP plate
( ) LCP plate
( ) LC DCP plate
( ) Pre-contoured locking plate
( ) Reconstruction plate
( ) One-third tubular plate
( ) Other type of surgical treatment. Please specify below:
___________________________________________________________
12. When a plate synthesis is performed, which is the most frequently used position for the placement of the plate?
( ) I only perform NON-operative treatment
( ) I do not use plates
( ) Anterior
( ) Superior
( ) Anteroinferior
( ) Other: _______________________________
13. For how long do you recommend immobilization after surgery?
( ) I only perform NON-operative treatment
( ) I do not immobilize the patient after surgery
( ) Immobilization at the discretion of patients and only for their comfort
( ) One week after the surgery
( ) One to two weeks
( ) Two to three weeks
( ) Three to four weeks
( ) Four to six weeks
( ) More than six weeks

## APPENDIX 2

**Table:** QUESTIONNAIRE FOR THE LATIN AMERICAN SOCIETY – EXCLUDING BRAZIL

Instructions:
Only one valid answer to each question, except question 5.
Consider the following conditions as a pattern for all questions.
**Treatment of CLOSED MIDSHAFT clavicle fractures in ADULT and CLINICALLY STABLE patients, with NO other fractures (excluding floating shoulder) or associated lesions (vascular and/or neurological)**
1. Years of work experience in orthopedics:___________
2. Country
( ) Argentina
( ) Bolivia
( ) Chile
( ) Colombia
( ) Costa Rica
( ) Ecuador
( ) El Salvador
( ) Guatemala
( ) Mexico
( ) Nicaragua
( ) Paraguay
( ) Peru
( ) Uruguay
( ) Venezuela
3. Type of work:
( ) Associated with an education institution
( ) No association with education institution
4. What radiographic classification do you prefer to use?
( ) Fracture morphology (fracture anatomy)
( ) Allman classification
( ) Robinson classification
( ) AO/OTA classification
( ) I do not use any classification
( ) Other classification: _____________________________________
5. Which of the following criteria would lead you to indicate a surgical treatment? (THIS IS THE ONLY QUESTION THAT ALLOWS MORE THAN ONE ANSWER).
( ) All my patients are treated with NON-operative treatment (if you choose this alternative, go to question 6)
( ) All my patients are treated with surgical treatment (if you choose this alternative, go to question 6)
( ) Displaced fracture WITH cortical contact
( ) Displaced fracture WITHOUT cortical contact (>2cm)
( ) Fracture shortening (>2cm)
( ) Comminuted fracture (>3 fragments)
( ) Segmental fracture
( ) Imminence of skin exposure
( ) Significant clinical deformity (aesthetic deformity)
6. For patients who are NOT surgically treated, what type of immobilization do you use?
( ) I only perform surgical treatment
( ) I do not use any type of immobilizer
( ) Simple arm sling in most cases
( ) Figure-of-eight bandage in most cases
( ) A combination of sling and figure-of-eight bandage in most cases
( ) Other type of immobilization. Please specify below:
___________________________________________________________
7. For patients treated NON-operatively using immobilizer, for how long do you continue the treatment?
( ) I only perform surgical treatment
( ) Up to two weeks
( ) Two to three weeks
( ) Three to four weeks
( ) Four to six weeks
( ) More than six weeks
8. For surgical treatment, what is the preferred synthesis used in spiral fractures?
( ) I only perform NON-operative treatment
( ) Kirschner wire
( ) Flexible intramedullary nail
( ) DCP plate
( ) LCP plate
( ) LC DCP plate
( ) Pre-contoured locking plate
( ) Reconstruction plate
( ) One-third tubular plate
( ) Other type of surgical treatment. Please specify below:
___________________________________________________________
9. For surgical treatment, what is the preferred synthesis used in oblique fractures?
( ) I only perform NON-operative treatment
( ) Kirschner wire
( ) Flexible intramedullary nail
( ) DCP plate
( ) LCP plate
( ) LC DCP plate
( ) Pre-contoured locking plate
( ) Reconstruction plate
( ) One-third tubular plate
( ) Other type of surgical treatment. Please specify below:
___________________________________________________________
10. For surgical treatment, what is the preferred synthesis used in transverse fractures?
( ) I only perform NON-operative treatment
( ) Kirschner wire
( ) Flexible intramedullary nail
( ) DCP plate
( ) LCP plate
( ) LC DCP plate
( ) Pre-contoured locking plate
( ) Reconstruction plate
( ) One-third tubular plate
( ) Other type of surgical treatment. Please specify below:
___________________________________________________________
11. For surgical treatment, what is the preferred synthesis used in complex fractures?
( ) I only perform NON-operative treatment
( ) Kirschner wire
( ) Flexible intramedullary nail
( ) DCP plate
( ) LCP plate
( ) LC DCP plate
( ) Pre-contoured locking plate
( ) Reconstruction plate
( ) One-third tubular plate
( ) Other type of surgical treatment. Please specify below:
___________________________________________________________
12. When a plate synthesis is performed, which is the most frequently used position for the placement of the plate?
( ) I only perform NON-operative treatment
( ) I do not use plates
( ) Anterior
( ) Superior
( ) Anteroinferior
( ) Other: _______________________________
13. For how long do you recommend immobilization after surgery?
( ) I only perform NON-operative treatment
( ) I do not immobilize the patient after surgery
( ) Immobilization at the discretion of patients and only for their comfort
( ) One week after the surgery
( ) One to two weeks
( ) Two to three weeks
( ) Three to four weeks
( ) Four to six weeks
( ) More than six weeks
